# Intubation in children presenting with seizures to a pediatric emergency department in a safety net hospital

**DOI:** 10.3389/fped.2026.1807879

**Published:** 2026-05-04

**Authors:** Mugdha Mohanty, Zakir Shaikh, Hovra Zahoor, Shivangi Kataria, N. Paul Rosman, Alcy R. Torres

**Affiliations:** 1 UMass Memorial Medical Center, Worcester, MA, United States; 2 University of Massachusetts Chan Medical School, Worcester, MA, United States; 3 HCA Florida Orange Park Hospital, Orange Park, FL, United States; 4 Syneos Health Inc, Morrisville, NC, United States; 5 Boston University Chobanian & Avedisian School of Medicine, Boston, MA, United States

**Keywords:** adolecent, children, emergency department, intubation, seizure, status epilepticus

## Abstract

**Objective:**

Patients seen for uncontrolled seizures in a Pediatric Emergency Department (ED) often undergo tracheal intubation (TI). We looked at factors associated with TI in children in a safety net hospital.

**Methods:**

This was a single-center retrospective case series. Fifty-six patients who had ongoing seizures at the time of arrival to the pediatric ED, between ages 0–21 years, were analyzed for demographics, seizure duration, seizure types, indications for TI, antiseizure medication on discharge, and neurological sequelae.

**Results:**

Forty-six percent of patients (26/56) underwent TI. The most common type of seizures in the intubated group was a complex febrile seizure (27%). Forty-two percent were intubated for airway protection and 46% were intubated for respiratory failure.

**Conclusions:**

In all patients presenting to a Pediatric ED with uncontrolled seizures, those undergoing TI most often had complex febrile convulsions, seizures longer in duration (*p* = 0.036), and seizures needing poly-drug therapy for seizure control (*p* < 0.001).

## Introduction

Seizures in children are a common neurological disorder. The global incidence of epilepsy (recurring unprovoked seizures) in children ranges between 41 and 187 per 100,000 worldwide; the incidence is highest in the first year of life and then decreases with age (1). The incidence of seizures is much higher in resource-limited countries owing to higher rates of infection such as meningitis/encephalitis, other febrile illnesses, metabolic derangements, hypoxia, malnutrition, and limited access to healthcare. Because the incidence of seizures remains high in resource-equipped countries, seizures are a common cause of presentation to hospital EDs (2, 3). In the USA from 2006 to 2017, there were an estimated 6.86 million ED visits for children with seizures. Of these, 5.41 million (78.9%) were brought to a general ED and 1.45 million (21.1%) to a Pediatric ED (4).

Seizures are life-threatening and prolonged seizures (more than 30 min) can lead to damage of multiple body organs, cardiopulmonary arrest, permanent neurological damage, and death (5). Respiratory decompensation remains the most feared complication from seizures and may lead to intubation. Although most cases of status epilepticus can be controlled with standard antiseizure medication, 10%–20% of these cases remain refractory to initial pharmacological treatment and may require further treatment with medications like midazolam or propofol, the availability of which may be compounded by agent availability and consistency. Adherence to such protocols carries significant sedative side effects, including respiratory suppression (6, 7). In such circumstances, intubation is commonly used for airway protection (8).

The common indications for intubation for seizures in children are respiratory distress, persisting seizure activity, and prolonged loss of consciousness (8). Additionally, intubation may be indicated in children with underlying respiratory or cardiac disease, or in children who have experienced a previous cardiac arrest from seizures. Intubation in pediatric seizures is associated with several potential complications, including airway trauma, hypoxia, and hemodynamic instability (8). Also, it is important to note that intubation can hinder the ability of the clinician to evaluate whether epileptic activity has resolved, potentially prolonging a patient's stay in the hospital and resulting in over-management of the seizures (8). Careful monitoring and management of these complications are critical to ensure optimal patient outcomes. Additionally, long-term outcomes may be influenced by factors such as the underlying etiology of the seizure, the presence of comorbidities, and the duration of intubation8. In most regions of the world, access to a pediatric ICU is not available and thus, seizure-related airway compromise effectively becomes a terminal event.

## Materials and methods

### Study design and setting

This was a single-center retrospective observational study that assessed intubation in patients presenting with seizures to a Pediatric ED. Demographic and clinical data were collected between 2010 and 2020. This study was reviewed and approved by the Institutional Review Board of the Boston University Chobanian & Avedisian School of Medicine. The requirement for informed consent was waived due to the retrospective nature of the study and the use of de-identified data obtained from existing medical records.

Boston Medical Center is one of the largest safety-net hospitals in Boston, Massachusetts, and provides healthcare regardless of insurance status or ability to pay.

### Study population and eligibility criteria

All eligible children and young adults 21 years old or younger who presented to the Pediatric ED at Boston Medical Center with ongoing seizures at arrival during the study period were included. Patients whose seizure had stopped or those who were post-ictal were excluded. No *a priori* power calculation was performed because of the retrospective design of the study.

The hospital's electronic medical record was queried for patients with seizures and related terms (status epilepticus, febrile seizures, complex febrile seizure, intubation, and ED). Complex febrile seizure is defined as a seizure with any of the following: duration longer than 15 min, seizures with focal features, and febrile seizure recurrence within 24 h.

### Data collection and study variables

Data was collected on age of seizure onset, duration of seizure activity, seizure description, antiseizure medications given, prior diagnosis of seizures and/or epilepsy, underlying causes of seizure, sequelae, and duration of hospital stay (including intensive care unit admission), including days intubated. Based on whether patients were intubated, they were divided into intubated and control groups for statistical analysis and interpretation.

### Statistical analysis

Patients were compared according to intubation status. Descriptive statistics were used to summarize demographic and clinical characteristics, including seizure etiology, seizure duration, antiseizure medication burden, prior seizure disorder, and clinical outcomes. Comparisons between groups were performed using statistical methods appropriate for categorical and ordinal variables, including two-proportion tests, Fisher's exact test, and Cochran-Armitage trend tests, as applicable. Odds ratios (ORs) with 95% confidence intervals (CIs) were calculated to quantify the association between selected seizure duration and antiseizure medication categories and the odds of intubation. All statistical tests were two-sided, and a *p* value <0.05 was considered statistically significant. All analyses were performed in R (version 4.5.1). Given the retrospective design, relatively small sample size, and multiple subgroup comparisons, the analyses were considered exploratory. No multivariable analysis was performed because the available sample size and number of outcome events were considered insufficient to support stable adjusted modeling with multiple candidate predictors (14, 15).

## Results

### Study population and clinical characteristics

Table 1 summarizes the demographic and clinical characteristics of intubated and non-intubated patients. Age distribution was similar between groups, with nearly identical mean ages (6.97 ± 6.74 vs. 6.98 ± 7.26 years), and a predominance of males in both groups (62% vs. 57%). Shorter seizure duration (<5 min) was more frequent among non-intubated patients (43% vs. 19%), whereas intubated patients more commonly presented seizure durations between 11 and 30 min (19% vs. 7%) and 31 to 59 min (27% vs. 17%). Generalized tonic-clonic seizures were the most common semiology in both groups, particularly among intubated patients (65% vs. 57%). A prior seizure disorder was more frequently documented in non-intubated patients (50% vs. 27%), while fever status was similarly distributed across groups. LEV was the most frequently reported etiologic/treatment-related category in both groups, especially among intubated patients (42% vs. 27%).

**Table 1:** Demographic and clinical characteristics by group.

Variable	Intubated patients (*n* = 26)	Non-intubated patients (*n* = 28)
Age (years)		
Mean ± SD	6.97 ± 6.74	6.98 ± 7.26
Median (Q1–Q3)	4.45 (1.73–11.75)	3.00 (1.50–14.25)
Sex		
Female	38%	43%
Male	62%	57%
Seizure duration		
<5	19%	43%
5–10	15%	17%
11–30	19%	7%
31–59	27%	17%
≥60	0%	10%
Unknown	19%	7%
Semiology		
GTC	65%	57%
Focal	15%	20%
Focal/CPS	4%	10%
Focal/GTC	4%	0%
Focal/MTL	0%	3%
Focal/Status	4%	0%
GTC + Focal	0%	10%
Unknown	8%	0%
Prior seizure disorder		
N	73%	50%
Y	27%	50%
Fever		
Febril	46%	50%
Afebril	54%	50%
Etiology		
LEV	42%	27%
None	35%	40%
OXC	8%	3%
PHT	8%	0%
LEV/OXC	4%	3%
LEV/OXC/PHT	0%	3%
LEV/VPA, VPA/LEV, Clorazepate	0%	3%
LTG	0%	3%
TPM/OXC	0%	3%
ZNS	0%	3%
Unknown	4%	10%

Fifty-six patients in total presented to the Pediatric ED, of which 52 had a known cause of seizure (93%). Twenty-six of the 56 patients were intubated (46%), whereas 30 were not intubated. Causes for seizures included complex febrile seizures, seizures due to non-adherence with antiseizure medication in a child with previous seizures, seizures in the setting of CNS infection, seizures from suicidal ingestion of drugs/medication, seizures accompanying a stroke, seizures caused by head trauma, and seizures of unknown causation.

The most common cause for seizures in the intubated group was a complex febrile seizure (7/26). Out of the seven patients with complex febrile seizures, six were complex by virtue of duration and the remaining one was classified as complex due to focal features. Additionally, one of the six patients with prolonged febrile seizures also had two febrile seizure recurrences in less than 24 h. In the intubated group, 8/26 patients had a pre-existing diagnosis of a seizure disorder and the most common indication for intubation was respiratory failure (12/26) (46%). Most intubated patients (18/26, 69%) remained intubated for one to two days. In the control group, 17/30 (57%) patients had a pre-existing diagnosis of a seizure disorder.

### Etiology and intubation

Patients whose cause of seizure was unknown were excluded from the etiology-specific comparative analysis. No significant difference was found in intubation when comparing complex febrile seizures with other causes of seizures (*p*-value = 0.849) (OR: 0.89; 95% CI: 0.26–3.09). By contrast, a significant difference in intubation was detected when comparing seizures in the setting of CNS infection with other causes of seizures (*p* = 0.023); the corresponding odds ratio was not estimable because of a zero cell. A significant difference in intubation was also detected when comparing patients with a previously diagnosed seizure disorder with other causes of seizure (*p* = 0.017) (OR: 0.21; 95% CI: 0.05–0.80).

### Antiseizure medication burden and intubation

Twelve out of 56 patients required no antiseizure medication for controlling seizures. The remaining 44 subjects were given one or more antiseizure medications. Only one patient who was intubated did not require any antiseizure medication due to resolution of seizure without use of antiseizure medication. Half of the patients (26/52) required two or more antiseizure medications to stop the seizure. Importantly, patients who required a greater number of antiseizure medications were more likely to undergo intubation. Six patients who received four or more antiseizure medications were intubated. A significant increasing trend was observed between the number of antiseizure medications administered and the need for intubation (*p* < 0.001).

Patients receiving only one antiseizure medication dose or no medication at all for controlling seizures had 89% reduced odds of intubation (OR: 0.11; 95% CI: 0.03–0.39) when compared with patients receiving two or more doses of antiseizure medications. Patients receiving two or fewer medications for the management of their seizures had 98% reduced odds of intubation (OR: 0.02; 95% CI: 0.002–0.157) when compared to subjects receiving three or more medications. These findings indicate that lower antiseizure medication burden was associated with lower odds of intubation, whereas intubated patients more frequently required multiple medications.

### Seizure duration and intubation

There were 49/56 patients who had a reported duration of seizures. Twenty-one of the 49 patients (43%) were intubated. The most common duration of overall seizure activity in the intubation group was 30–60 min (7/26) followed by 10–30 min (4/26). A significant increasing trend was observed between seizure duration and intubation (*p* = 0.038).

Seizure duration was categorized as less than 5 min, 5–10 min, 10–30 min, and 30–60 min. Three patients with seizures lasting 60 min or more did not undergo intubation because their seizures were a combination of focal and generalized seizures. Sometimes starting as focal seizure with subsequent generalization and some other times a generalized seizure followed by focal features which decreased the risk of cardiorespiratory complications as expected in long GTC seizures. For inferential analyses, seizure duration categories were additionally grouped as less than 10 vs. 10–30 min and less than 10 vs. 10–60 min.

A duration of seizure activity of 10–30 min placed the patient at 7.8 times the odds of intubation (95% CI: 1.1–41.0) vs. seizures less than 10 min in duration (*p* = 0.042). Overall, a longer duration of seizure activity (10–60 min) placed the patient at 4.2 times the odds of intubation (95% CI: 1.2–14.4) compared to seizures under 10 min in duration (*p* = 0.036). Other comparisons between seizure duration and odds of intubation were not statistically significant.

The main factors associated with intubation are summarized in Table 2. For transparency, detailed patient-level clinical presentation, management, and outcomes for the intubated and non-intubated groups are presented in Tables 3 and 4, respectively.

**Table 2:** Summary of factors associated with intubation.

Domain	Category/comparison	Main result	Effect estimate	*p*-value
Seizure etiology	Complex febrile seizure vs. other known causes	No significant association with intubation	OR: 0.89 (95% CI: 0.26–3.09)	0.849
	CNS infection vs. other known causes	Significant association with intubation	OR not estimable because of a zero cell	0.023
	Previously diagnosed seizure disorder vs. other seizure causes	Significant difference in intubation	OR: 0.21 (95% CI: 0.05–0.80)	0.017
Antiseizure medication burden	Increasing number of antiseizure medications	Significant increasing trend in intubation	Not applicable for global trend test	<0.001
	0–1 medication vs. ≥2 medications	Lower odds of intubation	OR: 0.11 (95% CI: 0.03–0.39)	0.0004
	≤2 medications vs. ≥3 medications	Lower odds of intubation	OR: 0.02 (95% CI: 0.01–0.16)	<0.001
Seizure duration	Increasing seizure duration	Significant increasing trend in intubation	Not applicable for global trend test	0.038
	10–30 min vs. <10 min	Higher odds of intubation	OR: 7.8 (95% CI: 1.1–41.0)	0.042
	10–60 min vs. <10 min	Higher odds of intubation	OR: 4.2 (95% CI: 1.2–14.4)	0.036

Unknown seizure etiology was excluded from etiology-specific comparative analyses. For descriptive purposes, seizure duration was categorized as <5, 5–10, 10–30, and 30–60 min; three patient with seizure duration ≥60 min was not intubated. For inferential analyses, seizure duration categories were additionally grouped as <10 vs. 10–30 min and <10 vs. 10–60 min.

**Table 3:** Clinical characteristics and outcomes of intubated patients.

Sex	Age, y	Sz dur, min	Semiology	Prior SD	F/A	Etiology	Disposition	LOS, d	ASM at discharge
M	18	<5	GTC	Y	Afeb	Hx stroke	Rehab	11–30	LEV
M	8	30–60	GTC	N	Feb	Viral ME	Home	11–30	LEV
F	4.9	30–60	GTC	N	Afeb	Epilepsy	Home	1–5	LEV
F	5	30–60	Unknown	N	Feb	Viral ME	Home	1–5	LEV
F	18	<5	Focal/CPS	Y	Afeb	Epilepsy	Home	1–5	OXC
F	0.8	10–30	GTC	N	Feb	Complex FS	Home	1–5	None
M	2.6	5–10	Focal	N	Feb	Feb	Home	6–10	None
F	0.5	10–30	Focal/GTC	N	Feb	Complex FS	Home	1–5	None
F	8	10–30	Focal	Y	Afeb	Epilepsy	Home	1–5	OXC
F	13	<5	GTC	N	Afeb	OD (TCA)	Home	6–10	LEV
M	15	Unknown	GTC	N	Afeb	TBI	Home	1–5	LEV
M	0.5	Unknown	GTC	N	Feb	Febrile	Home	1–5	None
M	2.5	10–30	GTC	N	Feb	Complex FS	Home	1–5	None
M	2.4	5–10	GTC	Y	Afeb	Epilepsy	Home	1–5	LEV
M	2.6	30–60	Focal/Status	N	Feb	Febrile	Home	1–5	LEV
M	0.7	5–10	GTC	Y	Feb	Complex FS	Home	1–5	None
M	1.8	<5	GTC	N	Feb	Complex FS	OSH	Unknown	Unknown
M	19	Unknown	GTC	N	Afeb	TBI	Rehab	11–30	PHT
M	21	30–60	GTC	Y	Afeb	TBI/Prior Epilepsy	AMA	1–5	PHT
M	1.1	30–60	GTC	N	Feb	Complex FS	Home	6–10	None
F	4	10–30	GTC	N	Feb	Complex FS	Home	1–5	None
M	1.7	30–60	GTC	N	Afeb	Epilepsy	Home	6–10	LEV/OXC
F	7	5–10	Focal	Y	Afeb	Epilepsy	Home	1–5	LEV
F	15	<5	GTC	N	Afeb	OD (Seroquel)	OSH	1–5	None
M	7	Unknown	Focal	N	Afeb	Epilepsy	Home	1–5	LEV
M	1	Unknown	Unknown	N	Afeb	TBI	Home	11–30	LEV

Sz, seizure; SD, seizure disorder; F/A, febrile/afebrile; LOS, length of stay; ASM, antiseizure medication; Afeb, afebrile; Feb, febrile; CPS, complex partial seizure; FS, febrile seizure; GTC, generalized tonic-clonic; ME, meningoencephalitis; OD, overdose; OSH, outside hospital; TBI, traumatic brain injury; LEV, levetiracetam; OXC, oxcarbazepine; PHT, phenytoin.

**Table 4:** Clinical characteristics and outcomes of non-intubated patients.

Sex	Age, y	Sz dur, min	Semiology	Prior SD	F/A	Etiology	Disposition	LOS, d	ASM at discharge
F	1.75	<5	Focal/CPS	N	Feb	Epilepsy	D/C Home	1–5	LEV
M	0.25	5–10	GTC	N	Feb	Unknown	D/C Home	Unknown	None
M	1	30–60	GTC	N	Feb	Complex FS	D/C Home	1–5	None
F	3	5–10	Focal/CPS	Y	Feb	Epilepsy	D/C Home	1–5	LEV
F	0.8	<5	Focal	Y	Feb	Complex FS	D/C Home	1–5	None
M	7	≥60	Focal/CPS	Y	Afeb	Epilepsy	D/C Home	1–5	LTG
M	1.5	5–10	GTC	N	Feb	Complex FS	D/C Home	1–5	None
F	1.75	<5	GTC	N	Feb	Febrile	D/C Home	1–5	None
M	1.6	<5	GTC	N	Feb	Epilepsy	D/C Home	1–5	TPM/OXC
M	1.5	<5	GTC + Focal	N	Feb	Complex FS	D/C Home	1–5	None
F	0.4	<5	Focal	Y	Afeb	Epilepsy	D/C Home	1–5	Unknown
M	18	Unknown	GTC	N	Afeb	Epilepsy	D/C Home	1–5	Unknown
F	17	30–60	GTC	Y	Afeb	Epilepsy	Unknown	Unknown	Unknown
F	19	5–10	GTC	N	Afeb	1st seizure	D/C Home	1–5	LEV
M	20	30–60	Focal	Y	Feb	Epilepsy	D/C Home	1–5	LEV/OXC
M	20	30–60	GTC	Y	Afeb	Epilepsy	D/C Home	1–5	LEV
M	19	<5	GTC	Y	Afeb	Epilepsy	D/C Home	1–5	LEV
M	16	<5	GTC	N	Afeb	1st seizure	D/C Home	1–5	None
F	16	Unknown	GTC	Y	Afeb	Epilepsy	D/C Home	1–5	LEV/VPA, VPA/LEV, Clorazepate
F	9	10–30	GTC	Y	Feb	Epilepsy	D/C Home	1–5	None
M	6	10–30	GTC	Y	Afeb	Epilepsy	D/C Home	1–5	LEV
M	5	≥60	GTC + Focal	Y	Afeb	Epilepsy	D/C Home	1–5	ZNS
M	3	≥60	GTC	N	Feb	Unknown	D/C Home	1–5	LEV
M	5.5	<5	GTC	N	Afeb	Complex FS	D/C Home	1–5	None
M	1.4	5–10	Focal	N	Feb	Complex FS	D/C Home	1–5	None
F	3	30–60	GTC	Y	Afeb	Epilepsy	D/C Home	1–5	OXC
F	0.1	<5	GTC + Focal	Y	Afeb	Epilepsy	D/C Home	1–5	LEV
F	0.4	<5	Focal/MTL	N	Feb	Complex FS	D/C Home	1–5	None
M	1.5	<5	Focal	N	Feb	Complex FS	D/C Home	1–5	None
F	6	<5	Focal	Y	Afeb	Epilepsy	D/C Home	1–5	LEV/OXC/PHT

Sz, seizure; SD, seizure disorder; F/A, febrile/afebrile; LOS, length of stay; ASM, antiseizure medication; Afeb, afebrile; Feb, febrile; CPS, complex partial seizure; FS, febrile seizure; GTC, generalized tonic-clonic; ME, meningoencephalitis; OD, overdose; OSH, outside hospital; TBI, traumatic brain injury; LEV, levetiracetam; OXC, oxcarbazepine; PHT, phenytoin.

## Discussion

Previous studies have investigated factors affecting intubation in patients presenting with seizures, especially seizures classified as status epilepticus. Rapid sequence intubation in the emergency room has been reported in approximately one in four children with status epilepticus (9). While there can be more than one factor leading to intubation, common contributors include epilepsy, underlying neurological disorders such as cerebral palsy, intracranial hemorrhage, severe traumatic brain injury, and developmental delay, any of which may impair the ability to clear airway secretions and increase the likelihood of airway compromise (4, 9).

The association between central nervous system (CNS) infections and intubation is greater when compared with other types of infections and has been described in studies performed in health systems with similar clinical characteristics (16). Patients with prolonged seizures have worse outcomes, and in settings where access to advanced airway management is limited, status epilepticus may become a terminal event (17). Other factors leading to intubation are comorbid respiratory disorders such as asthma in children, in which complicating seizures increase the risk of respiratory failure requiring airway intervention (9).

In clinical practice, apnea and respiratory failure often prompt early intubation, sometimes before adequate attempts at seizure control are made. This may reflect a clinical tendency to prioritize airway protection, either due to immediate respiratory compromise caused by the seizure or preemptively in anticipation of apnea associated with seizure activity or the administration of multiple antiepileptic drugs (AEDs) (11). A secondary analysis of the Established Status Epilepticus Treatment Trial (ESETT) demonstrated that practice pattern variation among centers influenced the likelihood of endotracheal intubation, suggesting that airway management decisions may be influenced not only by clinical severity but also by institutional practice patterns (11).

The high rate of intubation observed in our study (46%) likely reflects a clinical decision-making process in which airway protection is prioritized as a pre-emptive measure. Given that 43% of our patients were intubated specifically for airway protection, clinicians may intervene with tracheal intubation in anticipation of potential respiratory compromise rather than waiting for objective respiratory failure to manifest. This pre-emptive approach is particularly relevant when multiple antiseizure medications (ASMs) are administered, as the cumulative sedative effects of these medications are known to suppress respiratory drive. While studies suggest that earlier neurological recovery is associated with a lower need for intubation, these relationships may be influenced by confounding factors including seizure severity and treatment delays (11).

Our findings suggest that intubation in children presenting with uncontrolled seizures may be more closely related to markers of seizure refractoriness than to seizure etiology alone. Prolonged seizure duration and the need for multiple antiseizure medications were the factors most strongly associated with intubation in our cohort. This interpretation is consistent with recent evidence from Rosati et al., who showed that refractoriness in pediatric convulsive status epilepticus is influenced by treatment timing, adherence to guidelines, and etiology (18). Taken together, these findings suggest that timely seizure control may be critical not only for neurologic stabilization but also for reducing the need for tracheal intubation.

In our study, the most common cause of seizures in the intubated group was complex febrile seizures, particularly those that were prolonged in duration. Seizure duration is likely the key driver for intubation in this group. Clinicians may be more inclined to treat prolonged febrile seizures aggressively with multiple antiseizure medications because of concern for potential hypoxic brain injury associated with prolonged seizure activity.

Specific treatment-related issues were managed according to the established Boston Medical Center status epilepticus protocol. To ensure consistency in reporting across this pediatric cohort, medication doses were summarized as medians. Lorazepam was administered at a dose of 0.1 mg/kg, with a maximum of 4 mg for the initial dose. If seizure activity persisted beyond 5 min, a second dose was permitted. For patients who had already received abortive medication prior to arrival in the emergency department, the protocol directed loading with a first-line status medication. Similarly, if a patient was already receiving a maintenance antiseizure medication but the estimated dose was considered low, an additional dose of that specific agent could be administered (19).

Some seizure characteristics may predict the need for intubation. Patients presenting with generalized convulsive seizures have a greater likelihood of intubation compared with those with focal or non-convulsive seizures (9). Seizures, particularly generalized or prolonged seizures, can occasionally lead to transient autonomic instability, including apnea, even when seizures are not refractory (7).

In our study, seizures in the setting of central nervous system (CNS) infection and a pre-existing diagnosis of a seizure disorder were intubated more often than other causes. Additionally, the duration of seizures is another important factor that affects intubation. This was seen in our study, where a longer duration of seizure (10–30 min) was found to increase by 7.8 times the odds of intubation compared to seizures less than 5 min in duration suggesting that seizure refractoriness and duration as the primary drivers of intubation rather than seizure etiology alone.

One of the most important factors influencing intubation are medications given to control seizure activity. The current practice guidelines for the treatment of status epilepticus recommend the use of benzodiazepine (diazepam or lorazepam) as a first-line agent (8). Benzodiazepines carry the risk of sedating patients, and their improper use can lead to poor outcomes, including the need for intubation and prolonged hospitalization (8, 13). One such meta-analysis by Uppal et al. looked at data from both adults and children and found eight studies reporting excessive use of benzodiazepines, with pooled odds of 5.79 for adverse outcomes including respiratory depression, intubation and ICU admission (13). In our study, the median time for intubation from arrival to the ER was 26 min. Five of the patients were intubated within 15 min of arrival to the ER and another eight were intubated in the next 15 min. The median time of intubation was 26 min. Though our study was not designed to assess medication failure, these data suggested that 50% of patients were intubated because of medication failure. We believe the correct medications were used in all cases. Our study also found that using two or more antiseizure medications significantly increased the need for intubation.

Siefkes et al., in their retrospective analysis of seizure events transported by air to a tertiary medical center, reported that 61% of seizure events had not received recommended treatment and that these patients were three times more likely to undergo intubation (10). The most common treatment deviation noted in that study was administration of more than two doses of benzodiazepine or use of subtherapeutic weight-based dosing.

Another large multicenter study by Rosenthal et al. investigated the incidence of endotracheal intubation in 478 patients with established status epilepticus. Of Among 478 enrolled patients, 117 (24.5%) underwent intubation within 120 min of treatment initiation. The study suggested that early neurological recovery was associated with a lower need for endotracheal intubation in patients with established status epilepticus (11). Practice patterns differed widely among hospitals, highlighting the need for standardized treatment protocols and guidelines to improve neurologic outcomes for patients with status epilepticus (11).

Respiratory failure was the indication for intubation in 46% of our patients, while 42% were intubated primarily for airway protection. Chin et al. reported that inappropriate emergency management of status epilepticus, particularly administration of more than two benzodiazepine doses, increased the likelihood of respiratory depression requiring intensive care (12). Such difficulty was not observed in our cohort.

Some limitations of our study include a relatively small sample size, its retrospective design, and reliance on clinical documentation to determine indications for intubation. Furthermore, the decision-making process for intubation sometimes failed to include important preparatory steps such as medication preparation and team coordination. Review of 12 of the records in our study show that there were no documented cases with ASM administration prior to intubation. This may reflect a variability in clinical practice or a prioritization of airway protection over seizure management. This highlights the need for further investigation into whether early and appropriate seizure treatment could reduce the need for intubation and influence clinical outcome. Another limitation is the available information about the medications used by EMS during transport for all patients. Also, our study was carried out exclusively in a safety net hospital caring for an underprivileged and underserved population. A significant portion of patients in a safety net hospital are either uninsured or underinsured, and often face socioeconomic barriers such as limited access to primary care, language barriers, housing instability, and transportation difficulties. These factors might contribute to a lack of seeking medical treatment in a timely manner, non-adherence to prescribed medication, and increased ED utilization, any of which may have influenced the rate of seizure recurrence and intubation in this group.

## Conclusions

In this exploratory retrospective study of pediatric patients with uncontrolled seizures presenting to a Pediatric ED in a safety net hospital, tracheal intubation (TI) was required in nearly half of the cases (46%). While complex febrile seizures were the most frequent etiology in the intubated cohort, they were not independently associated with a higher likelihood of TI when compared to other seizure types. Instead, the primary drivers of intubation were markers of seizure refractoriness: prolonged seizure duration and requirement for multiple antiseizure medications. Specifically, patients receiving two or more anti-seizure medications had significantly higher odds of intubation, highlighting the critical interplay between aggressive pharmacologic management and respiratory compromise. Despite the sample size and single-center study, these findings suggest the need for standardized protocols that optimize early seizure termination while mitigating the risk of respiratory failure. Future prospective research is warranted to define optimal intubation thresholds and determine how timing of intervention influences long-term clinical outcomes in underserved populations.

## Data Availability

The raw data supporting the conclusions of this article will be made available by the authors, without undue reservation.
